# Open reduction and Internal Fixation of Displaced Proximal Humerus Fractures with AO Stainless Steel T-Plate

**DOI:** 10.5704/MOJ.1403.011

**Published:** 2014-03

**Authors:** S Hussain, MA Gul, SA Dhar

**Affiliations:** Hospital for Bone & Joint Surgery, Srinagar, India; Hospital for Bone & Joint Surgery, Srinagar, India; Sher-i Kashmir Institute of Medical Sciences, Medical College & Hospital, Srinagar, India

## Abstract

**Background:** Proximal humeral fractures are considered the last unsolved fractures in orthopaedics. The treatment is controversial and various operative modalities have been reported in the literature. The aim of the present study was to evaluate functional outcome and complication rate after open reduction and internal fixation of displaced proximal humerus fractures by proximal humerus AO stainless steel T-plate.

Twenty-five (25) patients with displaced proximal humerus fractures treated with proximal humerus T-plate between May 2005 and June 2008 were included in the study. Fractures were classified according to the Neer classification into displaced 2-part, 3-part, and 4-part fractures. Patients were followed-up for a minimum period of two years. Functional evaluation was done according to the Neer scoring system. Scores were compared with other studies in the literature using similar implant.

Twenty patients had 2-part fracture, four had 3-part fracture, and one had 4-part fracture. Eighty-eight [88% (n = 22)] patients had good to excellent result, eight [8% (n = 2)] had fair, and four [4% (n = 1)] had poor result. Difference in Neer’s score between 2-part and 3-part fractures was not significant. Complications encountered in this series were screw backout in 8% (n = 2), superficial infection in 12% (n = 3), and avascular necrosis in 4% (n = 1) of cases.

We conclude that proximal humerus AO T- plate is a cheap and easily available implant, aspects which are particularly relevant in third world countries like India. It gives reliable fixation for 2-part and 3-part fractures. Its use in more complicated fracture patterns of 4-part fractures is not recommended.

**Key Words:**

Proximal humerus fractures, proximal humerus stainless steel T-plate, unstable fracture

## Introduction

The proximal humeral fractures constitute 2-3% of all fractures^1^ and are the third most frequent fractures in elderly patients after hip and Colles’ fractures ^2^. Eighty-five percent of these fractures are undisplaced or minimally displaced and are effectively treated with immobilization in a shoulder immobilizer followed by early motion^3,4^. The remaining 15% of fractures are displaced and pose a therapeutic challenge. Most fractures of proximal humerus occur through osteoporotic bone in elderly patients, common mechanism being a fall on the out stretched hand^2^. In younger patients, high-energy trauma is more frequently involved. Threefourth of the fractures occur after the age of 60 years and women outnumber men 3:1^5^. Strong muscle contractions may lead to greater tuberosity fractures, as occurs in patients who suffer electric shock and seizures. About 7-15% of gleno-humeral dislocations have associated greater tuberosity fractures. Lesser tuberosity fractures tend to occur in association with posterior gleno-humeral dislocations.

While the literature strongly suggests non-operative treatment for undisplaced fractures^4^, the management of displaced fractures is still controversial and challenging. Though a wide variety of treatment modalities have been used including external fixation, internal fixation with Kirschner wires, transosseous suture fixation, tension band wiring, screw fixation, standard plate and screw fixation and hemi arthroplasty^6,7,8^, consensus is not available on the ideal treatment modality especially for 3-part and 4-part fractures^9^.

The aim of the present study was to evaluate functional outcome and complication rate after open reduction and internal fixation of displaced proximal humerus fractures with proximal humerus AO stainless steel T- plate.

## Materials and Methods

### 

**Fig. 1: Preoperative anteroposterior (1) and axillary (2) view radiographs of proximal humeral fracture.**
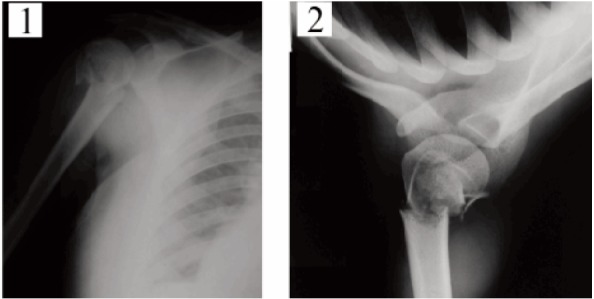


This was a prospective study conducted in our institute on a consecutive series of patients for the treatment of displaced^10^ (angulation of the articular surface of >45 degrees or displacement of more than 1 cm between the major fracture segments) 2-part, 3-part, and 4-part proximal humerus fractures from May 2005 to June 2008. Twenty-five (25) patients were included in the study. Closed fractures, 3-part and 4-part fractures and unstable 2-part fractures occurring in adults (age group of 18 years and beyond) in both sexes were included. Antero-posterior, lateral and axillary view xrays of the shoulder were obtained in all patients [Figure 1]. Three dimensional (3-D) CT reconstruction was used only in one patient with Neer type 4 fracture in whom head-splitting fracture was suspected. Fractures were classified according to the Neer classification^10^ into 2-part, 3-part, and 4-part [Table II]. There were twenty 2-part (80%), four 3-part (16%), and one 4-part (4%) fractures [Table I]. We used proximal humerus AO T-plate in all patients and contoured it with the help of a template.

The most commonly used classification of shoulder fractures is that of Neer (Neer 1970)^11^. Sidor et al^12^ demonstrated slightly improved mean interobserver reliability coefficient as well as mean intraobserver reproducibility for the Neer classification. Similar results were also observed in another study^13^. Although refinements^14,15^ and more detailed systems^16,17^ have been produced, none has gained the level of acceptance of the Neer classification^10^.

**Fig. 2: Posoperative radiograph showing good reduction.**
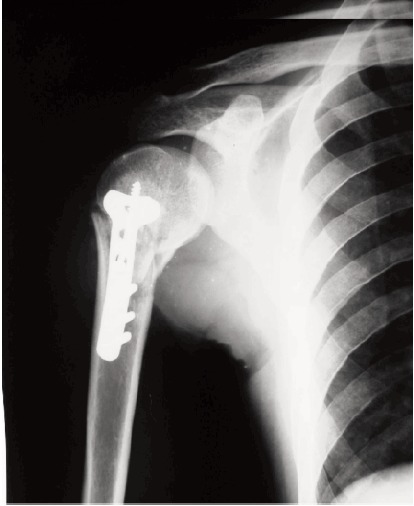


All the fractures were operated using a standard deltopectoral approach with the patient in the supine position on a radiolucent table with access for image intensifier to obtain intraoperative anteroposterior and axillary views. The long head of biceps acted as a guide to the interval between the greater and the lesser tuberosities. Fractures were reduced by correcting any varus or valgus deformity of the head fragment in relation to the shaft; gentle manual traction and manipulation with periosteal elevators and the fractures provisionally stabilized with multiple 2 mm K-wires. The Kwires could also be used as joy-stick to manipulate the head fragment. Tuberosity fractures were then reduced and fixed by means of non-absorbable Ethibond sutures whenever required. An AO plate was moulded to perfectly fit the lateral aspect of proximal humerus lateral to the tendon of the long head of biceps and 7-8 mm below the greater tuberosity. The screws were inserted usually in the proximal humeral shaft fragment initially. The head fragment was then gently manipulated and reduced as anatomically accurately as possible. Postoperative x-rays were obtained in all patients [Figure 2].

**Fig. 3: Follow-up radiograph at 24 months.**
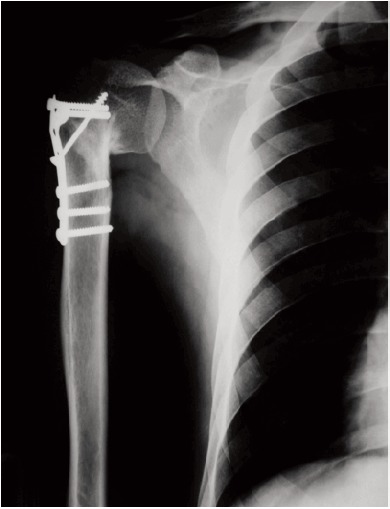


Postoperatively shoulder immobilizer was applied for two weeks and arm sling till fracture union. Passive-guarded physiotherapy was started in the first postoperative week and continued till fracture union. Active range of motion exercises and passive stretching exercises were started after fracture union. Patients were followed up at 6 weeks, 3 months, 6 months, 1 year, 1.5 years, and 2 years [Figure 3]. At each visit, functional evaluation was done according to the Neer scoring system [Table II]10 that included the following; (1) pain, (2) functional ability (Strength, reaching, stability), (3) range of motion and (4) anatomical features (rotation, angulation, retracted tuberosity, avascular necrosis etc.). The Neer score was graded as poor (<70 points), fair (70-79), good (80-88), or excellent (>89).

## Results

A total of twenty five (25) patients with proximal humerus fractures were treated during the period of study. All twentyfive patients were available at final evaluation. Patient characteristics are represented in Table I. Patients were operated after an average period of 8 days. The average union time was 3 months (range 2-5 months). Clinical evaluation was done according to Neer score. Eighty-eight percent (n = 22) patients had good to excellent result, 8% (n = 2) - fair, and 4% (n =1) - poor result. Neer scores for 2-part and 3-part fractures were significantly superior to those of 4- part fractures. The lone 4-part fracture went on to develop avascular necrosis of the humeral head. The difference between 2-part and 3-part fractures was not significant. Overall 8 complications occurred in 4 patients [Table III]. Activity related pain occurred in four patients (16%). Three patients (12%) developed superficial wound infection. The infection subsided with daily antiseptic dressing and amikacin antibiotic wash. Two patients (8%) had screw backout from the humeral head during the post-operative rehabilitation. Active range of motion exercises were delayed in these patients. The fractures ultimately united but these patients had only fair results. One patient (4%) went on to develop avascular necrosis as described previously.

## Discussion

Treatment of displaced proximal humerus fractures has been controversial^18,19^. For displaced fractures, traditional treatment with conventional plates and screws has been associated with fairly satisfactory results and some complications^20^. The AO stainless steel T-plate achieves adequate and stable fixation particularly in younger patients, allows early range of motion exercises and minimizes chances of mal-union and non-union^21,22^.

In 1987, Kristiansen & Christensen reported in a series of 21 patients with proximal humeral fractures. Out of 8 patients with surgical neck factures treated with ORIF, 3 had excellent results, 2 had good results and 3 had poor results. In 8 patients with greater tuberosity fractures 3 had good results, 2 had fair results and 3 had poor results^23^.

Rudolf S et al in 1990 reported 143 patients with proximal humeral fracture treated with internal osteosynthesis over a period of 10 years (1978-1988). They observed that the Tplate has a number of advantages because it was thinner and could be contoured to the surface of the bone and sized to fit. They emphasized on rigid fixation to minimize pain which allows earlier functional active therapy, thereby providing better long term result^24^.

SK Moda et al in 1990 reported open reduction internal fixation in^25^ severely displaced fractures and fracturedislocations of the proximal humerus. In 15 patients AO Tplate was used and in 10 a semi-tubular plate as a blade plate.Overall results were excellent and good in 84% patients. They attributed the successful results in part to avoidance of technical errors like high positioning of the plate, an unstable osteosynthesis, and penetration of joint by screws^25^.

In 1994, Rene D Esser reported a series of 31 patients with 3-part fractures treated with AO-plates and modified cloverleaf plates. Twenty-three had excellent results, 2 good results and 6 fair results^26^ .

Koval, Blair, Takei reported in 1996 that plates and screws provided significantly stronger fixation in fresh frozen specimen in fracture neck of humerus than other methods of fixation like Ender nail and tension band wiring^4^.

Koval KJ et al in 1996 performed a cadaver study to compare the stability and ultimate strength of 10 standard fixation techniques for the treatment of surgical neck fractures of the proximal humerus. The T-plate and screws provided significantly stronger fixation. The Ender nails/tension band construct was the second strongest fixation technique^20^.

We found no significant difference in outcome between patients less than 60 years of age and those above. One osteonecrosis occurred in our series in a 60-year old female with 4-part fracture involving mainly the anatomical neck. A comparison of the present study with other reported studies [Table IV] shows the comparative outcome where similar implants had been used.

The high percentage of good results in our series can be attributed to the following factors: 1. Younger age with good bone quality (average age 42.50); 2. Fracture type (80% two-part fractures); 3. Anatomical reduction in the majority of cases, and 4. Good patient compliance for postoperative rehabilitation.

Proper patient selection, good surgical technique and supervised physiotherapy are of paramount importance to achieve good results.

The technical points to be remembered during open reduction and internal fixation of displaced proximal humeral fractures include:- 1. The plate must be placed at least 7-8 mm below the greater tuberosity to avoid impingement on abduction; 2. The screws must not penetrate the humeral head into the joint 3. The surgeon must strive for as anatomical a reduction as possible and stable osteosynthesis 4. Early and supervised rehabilitation in association with rehabilitation specialists is important for translating good surgical technique into best results.

**Table I: Patient details and type of fracture:**
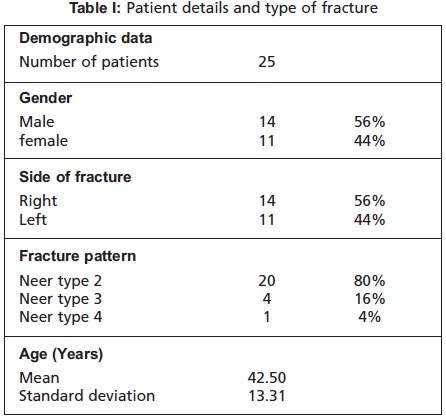


**Table I: Functional assessment key and scoring system by Neer (1970):**
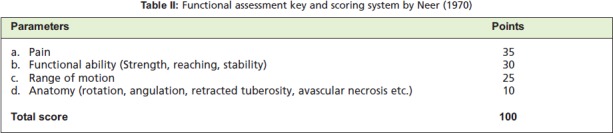


**Table I: Complications associated with open reduction and internal fixation of proximal humeral fractures with AO T-plate:**
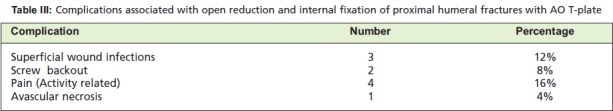


**Table I: Comparison of results amongst multiple studies:**
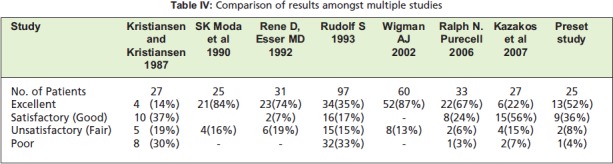


## Conclusion

We conclude that proximal humerus T- plate fixation for 2- part and 3-part fractures has good functional outcome. Use of this implant is technically less demanding and most of the complications occur because of intraoperative technical errors. The plate is cheap, easily available and requires simple instruments for application that are available in a third world setting like India. Its use in elderly patients (> 60 years age) and 4-part fractures is associated with high complication rate.
